# Transcriptional features of genomic regulatory blocks

**DOI:** 10.1186/gb-2009-10-4-r38

**Published:** 2009-04-19

**Authors:** Altuna Akalin, David Fredman, Erik Arner, Xianjun Dong, Jan Christian Bryne, Harukazu Suzuki, Carsten O Daub, Yoshihide Hayashizaki, Boris Lenhard

**Affiliations:** 1 Computational Biology Unit, Bergen Center for Computational Science, and Sars Centre for Marine Molecular Biology, University of Bergen, 5008 Bergen, Norway; 2 RIKEN Omics Science Center, RIKEN Yokohama Institute, 1-7-22 Suehiro-cho, Tsurumi-ku, Yokohama, Kanagawa, Japan; 3 Current address: Department for Molecular Evolution and Development, Centre for Organismal Systems Biology, Faculty of Life Sciences, University of Vienna, Althanstrasse, 1090 Wien, Austria

## Abstract

**Background:**

Genomic regulatory blocks (GRBs) are chromosomal regions spanned by highly conserved non-coding elements (HCNEs), most of which serve as regulatory inputs of one target gene in the region. The target genes are most often transcription factors involved in embryonic development and differentiation. GRBs often contain extensive gene deserts, as well as additional 'bystander' genes intertwined with HCNEs but whose expression and function are unrelated to those of the target gene. The tight regulation of target genes, complex arrangement of regulatory inputs, and the differential responsiveness of genes in the region call for the examination of fundamental rules governing transcriptional activity in GRBs. Here we use extensive CAGE tag mapping of transcription start sites across different human tissues and differentiation stages combined with expression data and a number of sequence and epigenetic features to discover these rules and patterns.

**Results:**

We show evidence that GRB target genes have properties that set them apart from their bystanders as well as other genes in the genome: longer CpG islands, a higher number and wider spacing of alternative transcription start sites, and a distinct composition of transcription factor binding sites in their core/proximal promoters. Target gene expression correlates with the acetylation state of HCNEs in the region. Additionally, target gene promoters have a distinct combination of activating and repressing histone modifications in mouse embryonic stem cell lines.

**Conclusions:**

GRB targets are genes with a number of unique features that are the likely cause of their ability to respond to regulatory inputs from very long distances.

## Abstract 2

CAGE tag mapping of transcription start sites across different human tissues shows that genomic regulatory blocks have unique features that are the likely cause of their ability to respond to regulatory inputs from very long distances.

## Background

**Figure 1:**
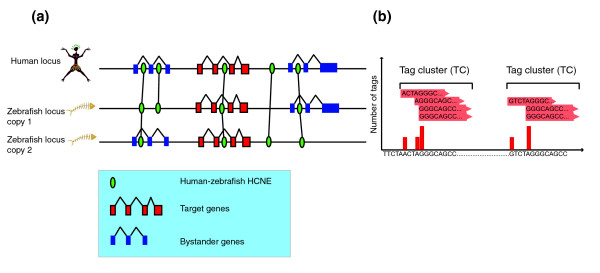
Definition of key terms. **(a) **The genomic regulatory block model and its possible evolutionary fate after whole genome duplication. Many HCNEs act as long-range regulators of target genes, while having no effect on bystander genes. The target gene is kept in both zebrafish copies of the loci, along with HCNEs, whereas bystander genes are differentially lost. **(b) **Tag clusters (TCs) are defined as overlapping CAGE tags (red horizontal arrows). Each distinct CAGE tag start corresponds to a CTSS. CTSSs are shown as vertical bars in the bottom track with the height of each bar corresponding to the number of CAGE tags for that CTSS.

It has been demonstrated recently that the loci of many key developmental regulatory genes are spanned by arrays of highly conserved non-coding elements (HCNEs) [1,2]. Many of these HCNEs function as long-range enhancers [3,4], collaboratively contributing to specific regulation of given target genes [2-5]. We have shown that the regions of most anciently preserved synteny in vertebrates [6] and insects [7] are due to the requirement to keep such arrays of HCNEs in *cis *to their target genes. This has led us to formulate the concept of genomic regulatory blocks (GRBs), which are functional regulatory units on a chromosome that are spanned by HCNEs and contain the gene regulated by HCNEs (the target gene). Those HCNE arrays often span large genomic regions of low gene density (gene deserts), but are in other instances found in the introns of, or beyond, unrelated neighboring genes (which we will refer to as 'bystander genes') that are kept in synteny with the target gene by virtue of being intertwined with the target gene's regulatory elements: this synteny lock-in can be escaped by the bystander gene after whole-genome duplication and subsequent rediploidization as seen in teleost fish genomes [6,8] (Figure 1a). The functions and expression patterns of bystander genes are unrelated to those of the target gene, suggesting that they are unresponsive to the regulatory input of HCNEs [6,7,9].

GRB target genes are among the genes with the most complex spatiotemporal expression patterns during development and differentiation, and this is controlled by long-range regulatory interactions [4,5,10]. Zebrafish transgenesis assays [10] have shown that an enhancer trap that contains a reporter gene downstream of the core promoter of *gata2 *(a GRB target itself), when integrated anywhere into a GRB, responds to its long-range regulatory elements in the manner of the corresponding target gene. One of the fundamental unanswered questions about gene regulation in GRBs is what mechanisms underlie the differential responsiveness of promoters of target and bystander genes to long-range regulation. We have demonstrated recently that differential responsiveness in *Drosophila *may be due to different types of core promoters [7]. In the case of genes inhabiting vertebrate GRBs, the existence of an equivalent distinction on the level of core promoter sequence is not so obvious. Both the developmentally regulated target genes and the neighboring, broadly expressed bystanders have core promoters that, in most cases, lack a TATA box and overlap CpG islands - segments of genome that are rich in CpG dinucleotides as opposed to general depletion of CpG dinucleotides in the rest of vertebrate genome sequence [11,12]. These core promoters are of the 'broad' type [13], characterized by the absence of a well-defined single transcription start site (TSS); instead, the transcription from them can start from multiple sites within a range of several dozen to several hundred nucleotides.

In this work we set out to investigate general transcriptional initiation properties of genes in genomic regulatory blocks, including differences in expression and promoter structure between the target and bystander genes in GRBs, and to discuss possible underlying causes for their differential responsiveness. We approach this by analyzing the properties of CpG island promoters of target and bystander genes to discover key differences that might be related to their differential responsiveness to long-range regulation. To define promoter architecture and subtype, we accurately map the TSSs and promoters of human genes using CAGE (cap analysis of gene expression) tag data [14,15] from a number of different expression contexts (over 20 different tissues, including embryonic tissues) produced in two most recent FANTOM projects ([13,14] and [16]). CAGE tag data yield a large number of short sequence tags corresponding to 5' ends of capped PolII RNA transcripts [14]. CAGE tags mapped to the genome paint a picture of TSS usage in different expression contexts. We examine the TSS properties of GRB target genes and bystander genes, and investigate transcriptional initiation events across a number of tissue-specific libraries and one time-course differentiation experiment. The time series experiment we used for this consists of six time points between 0 and 96 h of phorbol 12-myristate 13-acetate (PMA)-stimulated THP1 cells, modeling macrophage differentiation [16]; it is the only CAGE-based time series experiment to date. The genome-wide histone acetylation data obtained in the same differentiation time-course allowed us to correlate the chromatin status of bystanders, targets and HCNEs with target gene expression. Our hypotheses can be summarized as follows: the apparently different responsiveness of GRB target genes and their immediate neighbors to (long-range) regulatory inputs will be reflected in the absence of correlation in expression between targets and bystander genes; HCNEs acting as enhancers of target genes at a particular point in time and space should have the corresponding chromatin domains in active state when they drive the expression of the target gene, which should be reflected by the presence of the corresponding epigenetic signatures; different responsiveness to long-range regulatory inputs will be reflected in different structural properties of the two classes of genes, that is, promoter organization and promoter sequence.

Our results reveal that target and bystander gene expression is decoupled by means of their different responsiveness to long-range regulatory inputs, and that expression of target genes, unlike bystanders, is significantly associated with acetylation of anciently conserved HCNEs within the corresponding GRB. Furthermore, GRB targets are encompassed by a high density of CpG islands and have a complex promoter structure with distinct motif content. These observations provide further insight into the HCNE mediated long-range regulation of genes at the core of the regulation of animal multicellularity.

## Results

### Promoters of GRB target genes have complex distribution of transcription start sites

We identified a set of 269 putative GRB target genes (see Materials and methods; Additional data file 1). A visual inspection of prominent GRB targets quickly revealed that they have a high density of tag clusters (TCs) around their start sites, determined in a 'conventional' way. We grouped CAGE tags into TCs when they overlapped by at least 1 bp and mapped to the same strand (see Materials and methods; Figure 1b); the goal of this clustering was for each cluster to correspond to an individual core promoter. Since many of the clusters were of the broad type [13], we chose the most frequently used CAGE TSS (CTSS) position (that is, the one supported by the highest number of tags) as the reference position of the cluster. TCs and their close surrounding genomic regions containing binding sites for the components of the pre-initiation complex (PIC) are regarded as core promoters; core promoters by definition do not contain tissue-specific or other context-specific binding sites, even though in rare known cases this may be possible. Individual, distinct TCs some distance apart are taken to correspond to separate, alternative promoters [13,17]. We then analyzed the distribution of TCs around four sets of genes. Set 1 comprises GRB target genes (see Materials and methods). Set 2 comprises bystander genes in GRBs; the comparison of their transcriptional properties in comparison with nearby GRB target genes is one of the main motivations for this study). Set 3 comprises other CpG island-overlapping genes outside GRBs; since most GRB target and bystander genes have CpG island-type promoters, genes elsewhere in the genome with the same general type of promoters should provide a general picture of their typical behavior). Set 4 comprises other (non-GRB, non-target) transcription factor (TF) genes; since most GRB target genes are TFs [1], this set serves as a control to exclude the possibility that certain transcriptional properties of GRB genes are actually general properties of TF genes.

**Figure 2:**
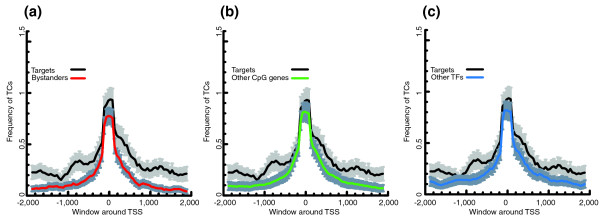
Density of TCs in target genes compared to bystander genes, other CpG genes and other TFs. Average TC density calculated in sliding windows of 250 bp over a 4,000 bp region for each gene set. Average TC densities with 90% confidence intervals of bystanders, other CpG island genes, and other TFs are compared with target genes. **(a) **TC frequencies of targets and bystanders. **(b) **TC frequencies of targets and other CpG genes. **(c) **TC frequencies of targets and other TFs.

**Figure 3:**
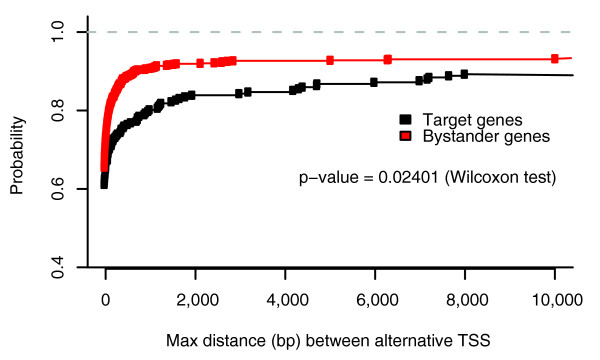
Cumulative distribution function of the maximum distance between distinct Ensembl TSSs covered by CAGE TCs for target and bystander genes. The distances for target genes are significantly larger (Wilcoxon test *p*-value = 0.0121) than those of bystander genes.

The average density of TCs in 4,000 bp windows centered on the most frequently used CTSSs revealed that GRB target genes have a wider distribution compared to bystander genes, other CpG island genes and other TFs (Figure 2). Similarly, GRB target genes had significantly higher TC counts in the 4,000 bp window around most used CTSSs (Wilcoxon test, *p*-value < 2.2e-16; Figure S1 in Additional data file 2). To ensure that this trend was not due to expression level difference between two sets of genes or fragmentation of the TCs due to undersampling or low expression, we compared the CAGE expressions in 4,000 bp windows around the most used CTSSs of target and bystander genes. We found that bystander gene expression was significantly higher than target gene expression (*p*-value = 0.0026, Wilcoxon test; Figure S2 in Additional data file 2). If lower expression of target genes caused undersampling of targets relative to bystanders, with resulting fragmentation of large target gene TCs into many smaller TCs, the average distance between adjacent TCs associated with target genes would be smaller compared to bystanders. However, the difference in distribution of distances was not significant (*p*-value = 0.07, Wilcoxon test; Figure S3 in Additional data file 2), and even showed a trend for distances between target gene TCs to be slightly larger. Therefore, lower expression of targets does not result in TC fragmentation artifacts, excluding this as a possible cause of the observed high number of TCs around target genes. Instead, the high density of TCs points to a possible higher usage of alternative promoters in GRB target genes. To confirm this hypothesis using an independent data source, we counted how many different Ensembl transcript start sites were covered by TCs, in bystander and target genes. Although GRB target genes did not have a significantly different number of distinct Ensembl start sites compared to bystanders (*p*-value = 0.149 Wilcoxon test), the maximum distance between distinct Ensembl start sites was significantly larger on average (Wilcoxon test, *p*-value = 0.0121; Figure 3). It seems that alternative promoters of the target genes are, on average, more widely spaced than those of bystanders. Combined with the fact that TCs of targets span a wider region around the most used CTSS (Figures 1 and 2), there might be more variation in the choice of alternative TSSs in target genes.

### GRB target gene promoters are characterized by a distinct collection of putative transcription factor binding sites

**Figure 4:**
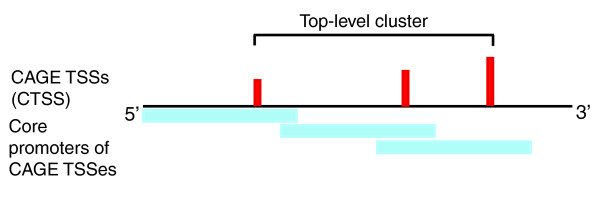
Definition of top-level clusters. Top-level clusters (top) were obtained by overlapping defined core promoter regions (-300, +100 bp) of CTSSs. If core-promoters of CTSSs overlap, they are clustered together. Blue regions denote core promoter regions for each CTSS (red bars).

In order to clearly define the extent of the TC density, and thus the extent of CTSSs, we clustered the CAGE tags by proximity (see Figure 4 for an illustration of this clustering approach). Our strategy differs from the original 'TC' clustering method in that it uses a distance threshold to define the extent of the cluster, rather than direct overlap of CAGE tags (see Materials and methods for details).

By mapping the clusters to genes, we concurrently defined the so called 'top-level promoter regions' for the genes. These promoters define alternative start sites whose core-promoters overlap with each other (Figure 4 and Materials and methods). We analyzed the transcription factor binding site (TFBS) content of these top-level promoters for bystander and target genes using JASPAR TFBS matrix profiles [18] (see Materials and methods). We also compared GRB target gene promoters with the set of promoters that overlap CpG islands but are not in a GRB or close to any region of high HCNE density (set 3 above). Our analysis indicated that GRB target promoters have an over-representation of homeobox, MADS and forkhead motifs (Table 1). However, all these motifs are AT-rich, which may not seem intuitive since both the target set and background sets are GC- and CpG-rich (see CpG results section). Although 93.6% of the target genes overlap with a CpG island, their most used promoters may not necessarily fully fall within those CpG islands. To check for such possible bias in AT composition, we performed a second comparison of target and background sets, but this time only considered promoters that were fully covered by CpG islands. Again, we found that the target set was enriched for similar AT-rich motifs such as Nkx2-5 and MEF2A in both comparisons (Table S1 in Additional data file 2). To further validate our results, we repeated the motif over-representation analysis using Clover [19], as well as the original over-representation method combined with phylogenetic foot-printing with mouse (Tables S2 and S3 in Additional data file 2) on the same background and target sets. Both approaches resulted in a similar set of over-represented AT-rich motifs, including Nkx2-5, FOXL1, and Pdx1. Taken together, these findings indicate genuine AT-rich motif enrichment in CpG-rich promoters of GRB target genes.

**Table 1:** Over-represented TFBSs in GRB target promoters

		Background: bystander gene promoters		Background: other CpG island promoters	
			
Family	Name	Hit *p*-value	Sequence occurrence *p*-value	Hit *p*-value	Sequence occurrence *p*-value
FORKHEAD	Foxa2	0.009	0.0001	0.0001	0.0001
FORKHEAD	Foxd3	0.0001	0.0001	0.0001	0.0001
FORKHEAD	FOXI1	0.0001	0.0001	0.0001	0.0001
FORKHEAD	FOXL1	0.0001	0.0001	0.0001	0.0001
FORKHEAD	Foxq1	0.0001	0.0001	0.0002	0.0001
ZN-FINGER, C2H2	Gfi	0.0068	0.0001	0.0001	0.0001
HOMEO	Lhx3	0.0003	0.0001	0.0001	0.0001
MADS	MEF2A	0.0001	0.0001	0.0008	0.0001
HOMEO	Nkx2-5	0.0001	0.0001	0.0001	0.0001
HOMEO	Nobox	0.0002	0.0001	0.0001	0.0001
HOMEO	Pdx1	0.0001	0.0001	0.0001	0.0001
HOMEO	Prrx2	0.0001	0.0001	0.0001	0.0001
ZN-FINGER, C2H2	RREB1	0.0001	0.0001	0.0001	0.0001
HMG	Sox5	0.0001	0.0001	0.0001	0.0001
HMG	SOX9	0.0025	0.0001	0.0005	0.0001
HMG	SRY	0.0001	0.0001	0.0001	0.0001
HOMEO	TCF1	0.0017	0.0001	0.0001	0.0001

TFBS content of target gene promoters was compared to two different background sets. The ones over-represented in both comparisons are output in the table. Two distinct *p*-values represent the significance of the number of profile hits in the target set (hit *p*-value) and the significance of the number of sequences having at least one hit in the target set (sequence occurrence *p*-value).

### GRB targets are often spanned by multiple long CpG islands with high CpG content

**Figure 5:**
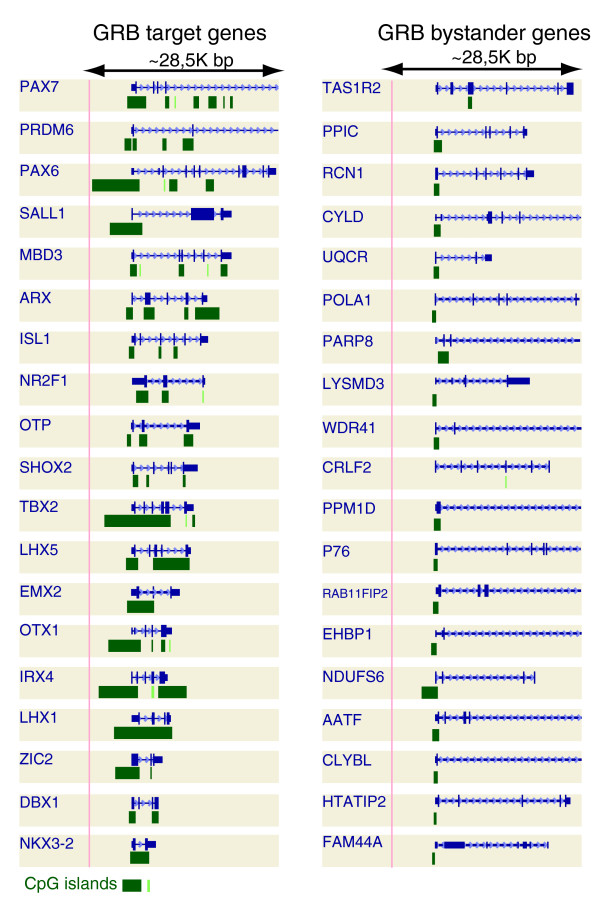
Examples of CpG islands covering target and bystander genes. Compared to bystander genes, target genes are typically covered by more and longer CpG islands (green rectangles). Genes are depicted in blue structures showing exon-intron configuration.

Inspection of GRBs in a genome browser quickly reveals that many GRB target genes overlap with long CpG islands, and often with several of them. These CpG islands are not limited to the 5' end of the genes, but also occur in introns or internal exons of the gene; in some cases, the entire target gene is spanned by one or several CpG islands (see Figure 5 for examples). Since most CpG islands normally map to promoters and are usually the targets of regulation by methylation [11,12,20], this arrangement is rather unusual. These CpG islands are the same ones recently shown to coincide with genomic regions bound by repressor Polycomb group proteins [21]. We mapped CpG islands to bystander genes, target genes and TFs not predicted to be GRB targets (gene set 4 described above), and compared their total CpG island length, count and CpG island length to gene length ratio; 94% of the GRB target genes and 75% of the bystanders overlapped with at least one CpG island.

**Figure 6:**
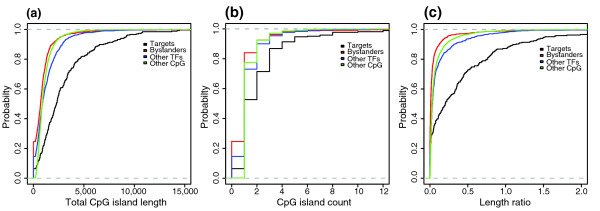
Cumulative distribution function plots for CpG island length, CpG island count and CpG island length to gene length ratio. In all of the plots, the values for target genes are significantly larger than other analyzed sets of genes (bystander genes, other CpG-covered genes and other TFs). **(a) **CpG scores for target genes and bystander genes. **(b) **CpG scores for target genes and other CpG island genes. **(c) **CpG scores for target genes and other TFs.

Furthermore, target genes have a significantly larger total CpG island length compared to bystanders, non-target TFs and other CpG island genes (all *p*-values << 0.05). The comparisons of CpG island count and CpG island length to gene length ratio showed similar differences (Figure 6; Table S4 in Additional data file 2).

**Figure 7:**
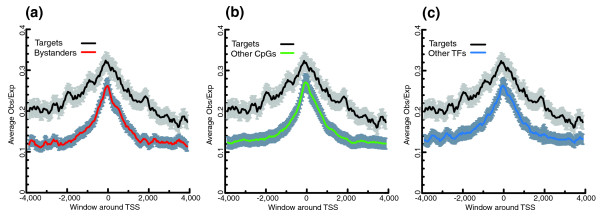
Average CpG scores for an 8,000 bp window around the most used TSS for targets, bystanders, other CpG genes and other TFs. CpG scores are significantly higher for target gene promoter regions than for background sets. The error bars indicate 90% confidence interval for the average scores. **(a) **Average CpG scores for targets genes and bystander genes. **(b) **Average CpG scores for targets genes and other CpG island genes. **(c) **Average CpG scores for targets genes and other TFs.

Also, the density of CpG dinucleotides was elevated around the most used TSS of the target genes. We calculated the observed/expected ratio of CpG dinucleotides, a standard method to predict CpG islands, for the [-4000,+4000] bp regions around the most used CTSS of target genes, bystander genes, other CpG islands and non-target TFs. In all comparisons, the CpG ratio in the window of interest was higher for target genes (Figure 7). This suggests there is an intrinsic difference in nucleotide composition of GRB target promoters compared to various backgrounds.

Another interesting feature of the GRB target promoters is that their corresponding mouse orthologs were mostly classified as 'bivalent promoters' in embryonic stem cells by Mikkelsen *et al*. [22]. Bivalent promoters have both repressive (H3K27me3) and active (H3K4me3) histone modifications in their promoter region. Around 70% (189 of 269) of GRB target orthologs have both repressive and active histone modifications in the reported system. Unsurprisingly, bivalent promoters are most often associated with developmental genes with high CpG density [22]. In contrast, only 13% of bystander gene orthologs are classified as bivalent (*p*-value < 2.2e-16, proportion test).

### Gene expression in GRBs: expression of target genes is uncorrelated to that of bystanders

Studies on individual target genes and their bystanders have shown that those genes have different spatiotemporal expressions during embryonic development [6,7]. At present, there is no suitable dataset to use for comprehensive quantitative comparison of target and bystander genes in developing embryos. However, since a subset of GRB target genes shows distinct temporal patterns in individual tissues and differentiating cell lines, such systems can still be used to assess expression correlation of targets and other genes in GRBs. The FANTOM4 project produced CAGE data and Illumina hg6v2 microarray expression data for one such time series [16]. We compared the expression profiles of target and bystander genes in three biological replicates of THP1 cells modeling macrophage differentiation in the 10-time-point time-course, spanning 0-96 h of differentiation of THP1 cells into macrophages *in vitro*. We found that 47% of the GRB target genes and 55% of bystanders were expressed in all the three replicates in at least one time point. However, when we correlated the expression profiles of targets and bystanders for each GRB, there were only 1% significantly correlated target-bystander pairs (Table 2). For this analysis, we only considered the correlation pairs that were significant in all replicates.

**Table 2:** Percentage of significant correlation coefficients for the THP1 macrophage differentiation time-course: expression profile comparisons between target and bystander genes

Replicate number	% of significant *p*-values	% of significant adjusted *p*-values
1	30.00	16.83
2	18.66	2.33
3	24.00	12.00
All	9.66	1.00

Expression pattern comparison between target genes and bystander genes for each replicate experiment are shown. Proportions of significant correlation coefficients are depicted in the cells.

An equivalent analysis for within-GRB bystander pairs showed that around 2% of the gene pairs had significantly correlated expression profiles (Table 3), but the correlation coefficient histograms (Figure S4 in Additional data file 2) show a trend toward a higher level of positive correlation, resulting in a left-skew (Table 4). This suggests that there is more positive correlation between bystander genes within a GRB than between the target gene and surrounding bystander genes. This was also evident when we compared the correlation coefficient distributions by a bootstrap Kolmogorov-Smirnov test or Wilcoxon test with the alternative hypothesis that bystander-bystander correlations were greater than target-bystander correlations. The bootstrap Kolmogorov-Smirnov test gave significant *p*-values for all replicates (Figure S5 in Additional data file 2). Furthermore, we checked the statistical relationship between target expression and bystander expression. The number of expressed and unexpressed bystanders was not significantly associated with expression of targets (*p*-value = 0.1928, Fisher's exact test; Table S5 in Additional data file 2).

**Table 3:** Percentage of significant correlation coefficients for THP1 macrophage differentiation time-course: expression profile comparisons between bystander gene pairs

Replicate number	% of significant *p*-values	% of significant adjusted *p*-values
1	31.6534	19.3908
2	19.7170	5.83554
3	22.6348	7.25022
All	9.01856	2.03359

Expression pattern comparisons between bystander gene pairs for each replicate experiment are shown. Proportions of significant correlation coefficients are depicted in the cells.

**Table 4:** Skewness of expression correlation distributions of GRB targets and bystanders for THP1 macrophage differentiation time-course

	Skewness Rep1	Skewness Rep2	Skewness Rep3
Correlation of bystander versus targets	-0.1962504	0.04708631	-0.06708631
Correlations of bystander with each other	-0.3967593	-0.1637775	-0.2637438

The skewness of correlation coefficient distributions for target-bystander gene pairs and for bystander pairs in the same GRB are shown. The skewness of distributions was calculated separately for each replicate. In all the cases, we observed more negative skew (left-skew) in bystander pair correlations.

To test the hypothesis that target genes are expressed in a time-specific manner, we examined the variation in expression level of target and bystander genes across the time-course experiment. If target genes tended to be expressed only at specific time points, this would be evident by larger variation of expression in the time-course experiment, compared to genes that were constitutively expressed. We found that targets expressed in macrophage-differentiation showed significantly higher variation than expressed bystander genes in all time-course replicates (replica 1 *p*-value = 2.144e-05, replica 2 *p*-value = 4.781e-05, replica3 *p*-value = 5.169e-06, Wilcoxon tests; Figure S6 in Additional data file 2).

### Acetylation status of HCNEs is associated with the expression of GRB targets during macrophage differentiation

For enhancers and promoters to be able to function, they must be accessible to TF proteins, which means they should be in a domain of open chromatin. H3K9 acetylation is considered a hallmark of open chromatin and, therefore, a requirement for promoter activity [23,24]. For the macrophage differentiation time-course described above, we analyzed H3K9 acetylation data for the 0 and 96th hours of differentiation to compare the acetylation state of target genes versus bystanders. First, we clustered acetylation sites into acetylation islands (see Materials and methods for details). Then, analogously to previous studies [23], we partitioned the genome into promoter, intergenic and intragenic regions. Next, we looked at the expressed and unexpressed GRB targets and whether or not they had promoter acetylation. As expected, the promoters of expressed GRB targets and bystanders were more frequently acetylated than those of unexpressed GRB targets and bystanders (two-sided Fisher's exact test *p*-value = 2.357e-12 for targets, and two-sided Fisher's exact test *p*-value < 2.2e-16 for bystanders; Tables S6 and S7 in Additional data file 2).

Next we analyzed the acetylation of human:zebrafish and human:chicken HCNEs, and found that GRBs of expressed targets contained one or more acetylated HCNEs more frequently than the GRBs of unexpressed targets (two-sided *p*-value = 0.0005741 for human:zebrafish HCNEs; *p*-value = 0.00125 for human:chicken HCNEs; Fisher's exact test; Tables S8 and S9 in Additional data file 2). In contrast, the presence of (one or more) acetylated HCNEs in the GRB was not associated with bystander gene expression, using the same test (Tables S10 and S11 in Additional data file 2). In addition, the proportion of acetylated HCNEs among all HCNEs in GRBs of expressed target genes was significantly higher than the similar proportion for GRBs of unexpressed targets (Zebrafish HCNE *p*-value= 1.545e-09 and chicken HCNE *p*-value= 1.326e-11, proportion test). Out of the acetylated HCNEs, 40% of human:zebrafish HCNEs and 34% of human:chicken HCNEs were intergenic. This indirectly shows that the acetylation status of both intergenic and intronic HCNEs is associated with the expression of the associated target gene.

## Discussion

This study provides a detailed survey of promoter properties of GRB targets and offers insight into their behavior during a differentiation time-course. GRB target genes show evidence of the existence of multiple promoters that span a large region when compared to several other gene sets. Multiple promoters might be instrumental in achieving the level of regulatory complexity characteristic of these target genes, which have the most complex spatiotemporal expression patterns of all metazoan genes [25,26]. Other striking features of the target genes are the long CpG islands that sometimes cover the whole gene (Figure 5), and a higher density of CpG dinucleotides around their most frequently used CTSS. Both the CpG island length and the existence of multiple promoters sets the target genes apart from other sets of genes, including genes in their immediate neighborhood with conserved synteny (bystander genes), other genes with CpG island promoters, and non-GRB target transcription factor genes.

Based on our previous whole-genome analyses [13], it is reasonable to expect a correlation between the number of TCs and CpG island length or CpG dinucleotide density, but the differences go deeper than that. Even though no CpG methylation is observed in *Drosophila *species, the increased CpG dinuclotide density trend has also been observed in developmental genes having promoters with stalled RNA PolII in *Drosophila melanogaster *embryos [27], the authors suggesting that stalling occurs in developmentally important genes only, in order to achieve rapid expression. Furthermore, the motif content of the target gene promoter regions is different from that of the bystanders and other CpG islands. We detected an over-representation of Forkhead family motifs along with Nkx2-5, MEF2A and SRY. Most of these motifs are bound by TFs that are GRB targets themselves. Forkhead TFs are major players in development: in the absence of Foxa2, mouse embryos can not develop further than embryonic day 8.5 and they lack notochord [28]. In addition, Nkx2-5 is essential for heart development [29] and MEF2A is required for somite development and hedgehog signaling in zebrafish [30] as well as vascular development in mammals [31].

Transgenesis [32] and enhancer trapping [6,10] experiments in zebrafish embryos showed previously that targets and bystanders have different spatiotemporal expression patterns. Here we have shown that the expression of target and bystander genes is also uncorrelated in a cell differentiation time course. Furthermore, we have provided support for the hypothesis that expressions of target genes are dependent on long-range enhancer input by showing that HCNEs having active chromatin domains are significantly associated with the expression of the target gene, consistent with being in an 'active' state and able to serve as regulatory inputs by binding TFs.

The distinct response of the target genes and their dependency on long-range regulation might be explained by the distinct motif content and sequence composition of their promoters. We showed recently that GRB target genes in *Drosophila *differ from the neighboring genes in the type and motif content of their core promoters [7], which might explain their differential responsiveness to long-range regulation. A similar mechanism, involving the motifs we have found to be over-represented in core promoters in this work, might play an analogous role in differential responsiveness in vertebrates. The existence of multiple promoters with multiple potential enhancers (HCNEs) suggests that there may exist many different promoter-enhancer pairings for the same gene. This might help achieve rapid activation by promoting expression from a number of promoters simultaneously or, alternatively, the high number of possible pairings may also provide robustness to the expression of target genes and allow for very precise and refined spatiotemporal patterns in different functional contexts. Since GRB target genes are central to developmental regulation, their expression should be robust and tightly coordinated even under varying external conditions. Most obviously, a large number of different promoter-enhancer pairings is needed because these genes have many different roles in time and space that require a complex switchboard of regulatory inputs arranged in a GRB.

## Conclusions

**Figure 8:**
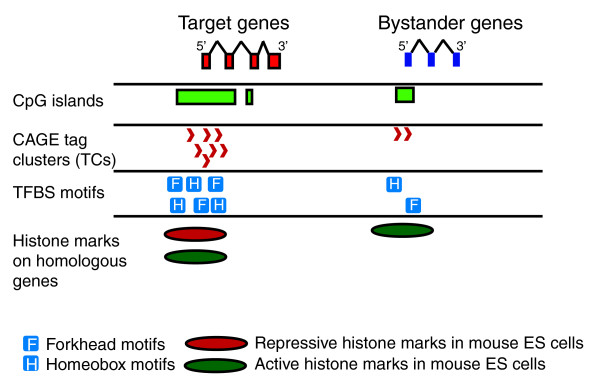
Illustration of main conclusions about properties of GRB target genes. Distinct tracks under the target gene and bystander gene models describe the properties of target genes in a comparative manner.

Target genes within genomic regulatory blocks have distinct properties when compared to their neighboring bystander genes and different background gene sets. These properties can be summarized as follows (Figure 8): wide TC distribution around the TSS, indicating possible multiple promoter usage; large CpG islands sometimes spanning the entire gene; distinct TFBS motif content; and mouse homologs of target genes having 'bivalent' histone marks.

While the exact molecular mechanism of differential responsiveness remains unknown, the above properties enable identification of key regulatory features of genes responsive to long range regulation by HCNEs and provide a guide to monitoring their activity on multiple levels. Additionally, GRB target gene regulation is different from bystanders and possibly dependent on input from HCNEs even in terminal myeloid differentiation.

## Materials and methods

### Determination of genomic regulatory blocks and their gene content

We designated the putative GRB target genes as the human TFs that were under a HCNE density peak and whose orthologs were in conserved synteny with an array of HCNEs in human:zebrafish alignments. Density peaks and syntenic regions were downloaded from the Ancora Genome Browser [33]. After retrieving the target genes, we located the overlapping human-zebrafish synteny blocks. Synteny blocks were defined by joined zebrafish (danRer5) to human (hg18) high scoring (level 1) net alignments obtained from the UCSC Genome Browser [34]. We joined neighbor net alignments if they were separated by at most 450 kb in human and 150 kb in zebrafish. If multiple synteny blocks overlapped with the target gene - for example, in the case of zebrafish paralogs - we took the union of those synteny blocks as the primary synteny block to be used in the analysis. Following this procedure, we retrieved all other Ensembl genes within those synteny blocks, and labeled them 'bystander' genes. For HCNE-acetylation analysis, we excluded GRBs that contained multiple plausible target genes with different expression status (see the section 'Acetylation site clustering and analysis' for details). Some of those excluded blocks harbored tandemly duplicated target genes, and in other cases two apparently separate GRBs could not be separated on the basis of zebrafish:human synteny.

### CAGE TC density in promoter regions

To calculate CAGE tag mapping densities, we combined FANTOM3 [14] and FANTOM4 CAGE tags. We only considered uniquely mapping tags, and during the clustering of tags into TCs we included clusters having at least one CTSS supported by at least two tags. For each TC we defined a representative location (supported by the highest number of tags per million). We calculated the density of TCs using a sliding window of 250 bp and a step size of 50 bp over a 4,000 bp region around the most used CTSS. We only considered TCs mapping to the sense strand of the gene. When calculating the error bars for targets we used sampling with replacement and sampled target set size samples 1,000 times and calculated the 90% confidence interval for the average TC density for each window. When calculating the error bars for other background sets (bystander genes, other CpG island genes and other TFs), we used sampling without replacement and sampled target set-sized samples again 1,000 times.

### CAGE tag clustering to top-level clusters and mapping to genes

The rationale of top-level clusters is to cluster CAGE tags based on the overlap of pre-defined core promoters. The clustering method is illustrated in Figure 4. First, in order to define top-layer TSS clusters for the genes, we excluded the singleton CTSS. Then, we extended each CTSS -300 bp and +100 bp. This corresponds to the core promoters for each CTSS. The overlapping core promoters of CTSSs mapping on the same strand formed so called 'top-level clusters'. Top-level clusters were mapped to genes in the following way. If the top-level cluster is in 500 bp proximity of a 5' end of an Ensembl transcript (Ensembl release 49 [34]) on the same strand, the top-level cluster/top-level promoter was mapped to that gene. If many top-level clusters were mapped to the same gene, we chose the one with the highest expression (number of supporting CAGE tags) as the representative one.

### Transcription factor binding site over-representation analysis

Putative TFBSs matching top-level promoters of bystanders and targets were extracted using Perl scripts and modules based on TFBS modules [35]. We used an 80% score cut-off and JASPAR position weight matrices when determining the hits. For each top-level cluster, we normalized the number of TFBS hits by the length of the cluster. We used a random sampling approach to assess the significance of the normalized total number of hits in target top-level promoters compared to bystander top-level promoters. We did this by randomly sampling target set-sized sets from a total set of promoters that included all promoters from the background sets (either bystander genes or other CpG island genes) and the target genes, and calculated the length-normalized total number of hits for each random set. We sampled 10,000 random sets with replacement and for each TFBS, we calculated the number of sets with equal or higher value to the original total hit count divided by 10,000. This gave the significance of the hits for each TFBS in the target promoter set. We also measured the significance of the number of sequences in the target promoter set having a certain TFBS motif. Again we used a random sampling approach with replacement to assess the significance. This time we counted the number of random sets that had a higher or equal number of sequences containing that TFBS hit. Again we calculated the *p*-value by dividing this number by the number of random sets. In order to call a TFBS motif in the target promoters significantly over-represented, both *p*-values had to be lower than 0.01. For the phylogenetic fooprinting approach, we extracted the orthologous mouse region for each promoter from the UCSC genome browser human-mouse NET alignment [36], and then searched the alignments for TFBS motifs using an 80% score cut-off and an 80% identity cut-off.

We also used the Clover [19] algorithm to detect TFBS motif over-representation. Clover is based on averaging likelihood ratios for sequences rather than counting motif hits. We considered motifs as over-represented in the target gene promoters compared to the bystander background set and the 'other CpG island gene' background set when the *p*-value was lower than 0.05 in both comparisons.

### CpG island and CpG score analysis of GRBs

We downloaded CpG island locations from the UCSC Genome Table Browser [36] and Ensembl gene boundaries from Biomart (Ensembl release 49) [37]. We extracted all CpG islands that overlapped with our genes of interest (target, bystander and other TF genes); additionally, we randomly selected 3,000 genes that were not GRB targets but overlapped CpG islands (other CpG island genes set). We removed the TFs that were also GRB targets from the set of other TFs. Our initial TF gene set was based on the set described in Vaquerizas *et al*. [38]. Afterwards, we calculated total length, number of CpG islands and CpG island length to gene length ratio for our four sets of genes (targets, bystanders, other CpG island genes and other TF genes). We compared these distributions using a Wilcoxon test (rank sum test) in R, testing for the alternative hypothesis that the true shift when comparing two given distributions was greater than 0.

CpG scores were calculated for a 250 bp window sliding in 50 bp steps over 8,000 bp regions around the most used CTSS. The error bars for CpG scores are calculated by sampling procedures, similar to the calculation of error bars of TC densities. CpG scores were the observed number of CpG dinucleotides divided by the expected number of CpG dinucleotides, as given by the following formula:



### Expression correlation analysis

We obtained normalized (quantile normalization between the arrays) Illumina expression data for a macrophage differentiation time-course [16]. There were ten time-points from 0 to the 96th hour of differentiation. For each probe, detection *p*-values were computed by BeadStudio software (Illumina). For the expression profile comparison analysis, we used a *p*-value cutoff of 0.05 for detection, and kept only the probes detected at nine or more time-points in each replicate. Using a less stringent threshold may result in genes that are detected in few time-points, which could compromise the correlation analysis. We assigned one probe for each gene using the probe annotation provided by the FANTOM4 consortium. When multiple detected probes mapped to one gene, we only considered the representative probe among them (as supplied by the microarray manufacturer), if any.

We calculated the correlation of expression for each target gene and its bystander genes; we tested the significance of correlation using the alternative hypothesis that the association was positive. We also calculated the correlations of bystanders with each other for each GRB. For comparison of bystander pairs, we excluded the pairs potentially sharing a bidirectional promoter, since we expect them to be co-regulated [39]. We defined bidirectional promoter genes as genes on the opposite strands that had an Ensembl TSS in 1,000 bp proximity of each other. For the correlation and significance tests we used the cor.test function in R. The *p*-values were corrected for multiple testing using Benjamini-Yekutieli false discovery rate correction from the multtest package in R.

### Expression variation analysis

We examined the relative expression variation of target genes compared to bystander genes using the same sets of genes as those used in the expression correlation analysis. We calculated the mean expression for each gene in each microarray replicate by averaging the normalized intensity values for all-time points, and computed the log2 of the ratio of normalized intensity to the mean expression. Then, we summed up the squares of those ratios to get the total relative variation for each gene. Following this, we compared the relative expression variation of target genes and bystanders for each replicate time-course experiment using a Wilcoxon rank sum test, with the alternative hypothesis that the variation in the target gene set was larger than in the bystander gene set.

### Selection of genes for acetylation analysis

We constructed two sets of genes for acetylation analysis, an expressed gene set and an unexpressed gene set. We decided to take the genes that had a detection *p*-value = 0.05 for both 0 h and 96 h as the expressed gene set, since acetylation data were available only for the 0 and 96 h time-points. Next, we extracted the genes that had no detected probes in any of the replicates at any time point, and used this set as the unexpressed gene set.

### Acetylation site clustering and analysis

H3K9 acetylation data were prepared using two biological replicates and two time points (0 h and 96 h) of the macrophage differentiation time-course using THP1 cells and ChIP-chip analysis. The acetylation regions with a *p*-value < 0.001 were clustered together into one if they were no more than 150 bp apart. The clustering was done for both time-points and separately for each replicate. The clustered acetylation sites were termed acetylation islands (AC islands). By including only AC islands that overlapped in each replicate, we prepared a stringent set for each time-point. Next, we made a unified stringent set for the 0 h and 96 h time-points by taking the union of AC island locations for both time points. In the end this gave only one set derived from two replicates and two time points. Since our expressed gene set consisted of genes expressed at both 0 h and 96 h, the expressed genes were expected to be acetylated in at least one time point.

For acetylation analysis we partitioned the genome into intergenic, intragenic and promoter regions. The promoter, in this case, was defined as 1,000 bp upstream and 1,000 bp downstream of the Ensembl TSS. The rest of the gene that was not part of the promoter region was categorized as intragenic. The parts of the genome that did not map to an Ensembl gene were categorized as intergenic. For expressed and unexpressed bystander and target genes, we counted the number of promoters and intragenic regions that had an AC island or not. We used these numbers to construct two-by-two tables for Fisher's exact tests, which we computed using the standard R function fisher test.

For the HCNE acetylation analysis, we downloaded human:zebrafish (minimum 70% identity over 50 bp) and human:chicken (minimum 90% identity over 50 bp) HCNEs from the Ancora Browser [33] and discarded HCNEs that overlapped with a promoter region (defined above). We then used the remaining HCNEs to count the occurrences of GRBs in which there was one or more HCNEs with an AC island at most 450 bp away. Existence of an H3K9 AC island does not necessarily mean that there is open chromatin in the exact position of the AC island. It is better interpreted as the occurrence of an open chromatin region in its proximity [23,24]. The degree of this proximity can be estimated from the CTSS and AC island relationship. AC islands usually do not overlap a CTSS. Most used CTSSs in the PMA time-course are observed to be, on average, approximately 450 bp away from an H3K9 AC island. The GRBs with and without acetylated HCNEs were divided further into GRBs of expressed targets and GRBs of unexpressed targets. When extracting the GRBs of unexpressed targets, we made sure that there were no other expressed target genes in the GRB; if there was, we excluded that region from the analysis.

## Abbreviations

AC: acetylation; CAGE: cap analysis of gene expression; CTSS: CAGE transcription start site; GRB: genomic regulatory block; HCNE: highly conserved non-coding element; TC: CAGE tag cluster; TF: transcription factor; TFBS: transcription factor binding site; TSS: transcription start site.

## Authors' contributions

BL and AA designed the study. AA, DF and JCB performed the analyses. HS, COD and YH designed and coordinated the FANTOM4 project and provided the CAGE and acetylation data. EA and XD provided data sets critical for the analysis. AA produced all the figures. AA, DF and BL wrote the manuscript with input from other coauthors.

## Additional data files

The following additional data are available with the online version of this paper: a spreadsheet listing the gene sets used in this study (Additional data file 1); supplementary tables and figures (Additional data file 2).

## Supplementary Material

Additional data file 1 Target genes, bystander genes, other CpG island genes (used for background) and other TF genes (used for background). Click here for file

Additional data file 2 Figures S1-S6 and Tables S1-S11. Click here for file
