# Supplementary material for: Transcriptional features of genomic regulatory blocks
Source: Genome Biol. 2009 Apr 19;10(4):R38. doi: 10.1186/gb-2009-10-4-r38 (PMC2688929; doi:10.1186/gb-2009-10-4-r38)
Supplement: Additional data file 2 — Figures S1-S6 and Tables S1-S11. [file gb-2009-10-4-r38-S2.doc]

# Supplementary Text

## Supplementary Figures


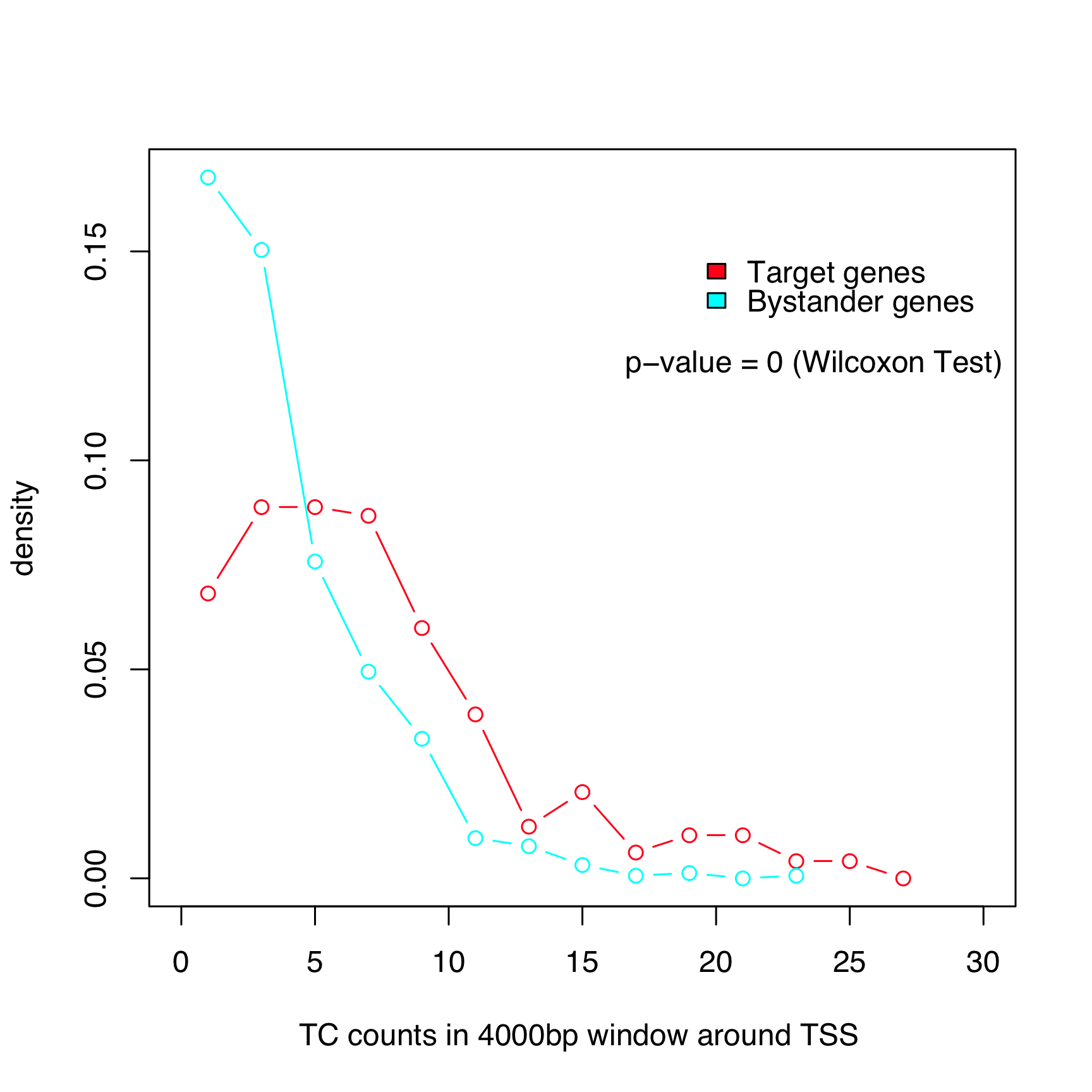


Figure S1. TC counts in 4000bp window around TSS for targets and bystanders

Histograms of target gene and bystander gene TC counts. Counts are coming from 4000 bp windows around the most used CAGE TSS for each target and bystander genes. Target genes have significantly (*p*-value < 2.2e-16, Wilcoxon Test) more TCs around their most used CAGE TSS, when compared using a Wilcoxon test.


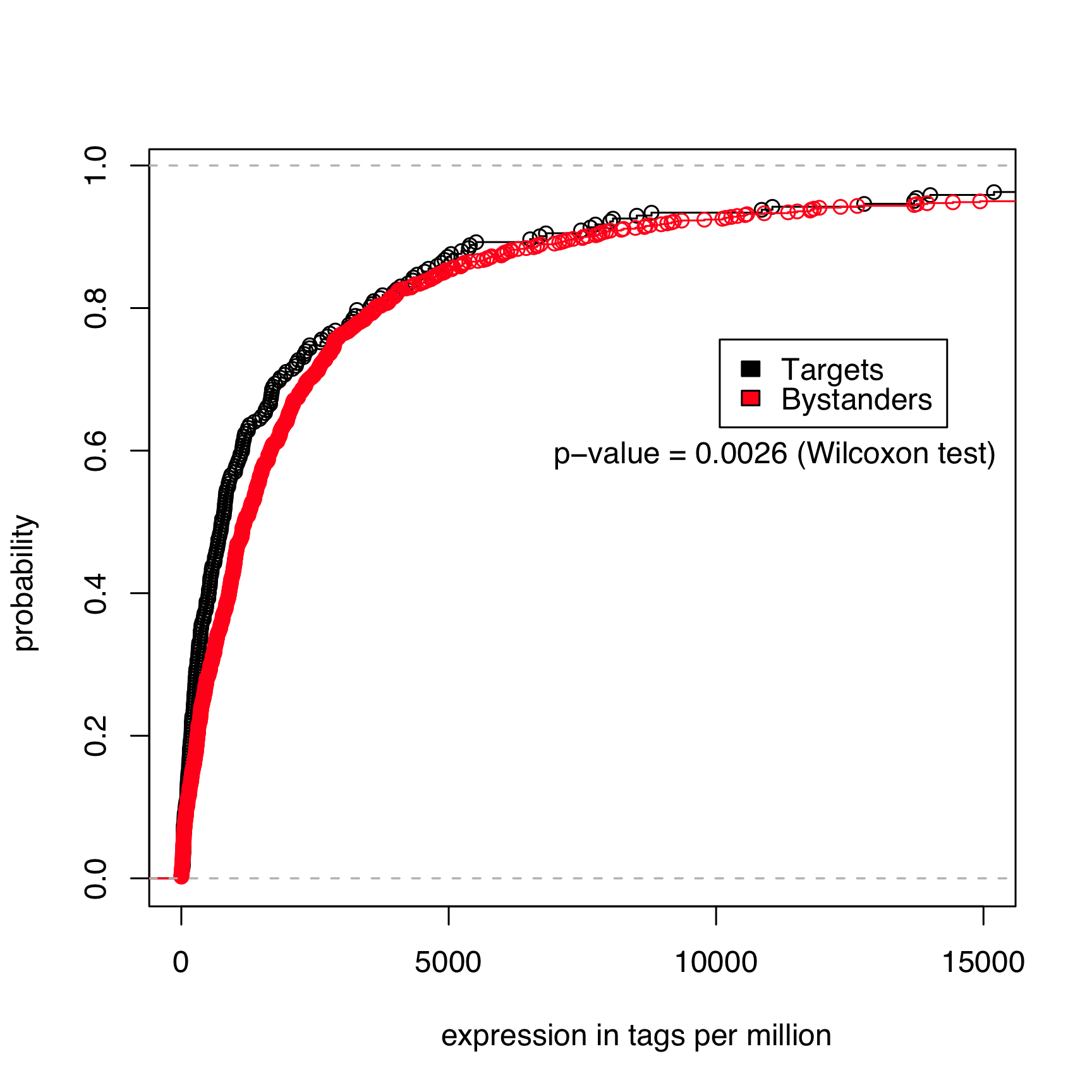


Figure S2. Cumulative density plot of CAGE expression

Cumulative distribution of CAGE expression for target genes and bystander genes as measured by summing the expression (tag per million) coming from every CAGE tag in a 4000 bp window around the most used CAGE TSS for each gene.


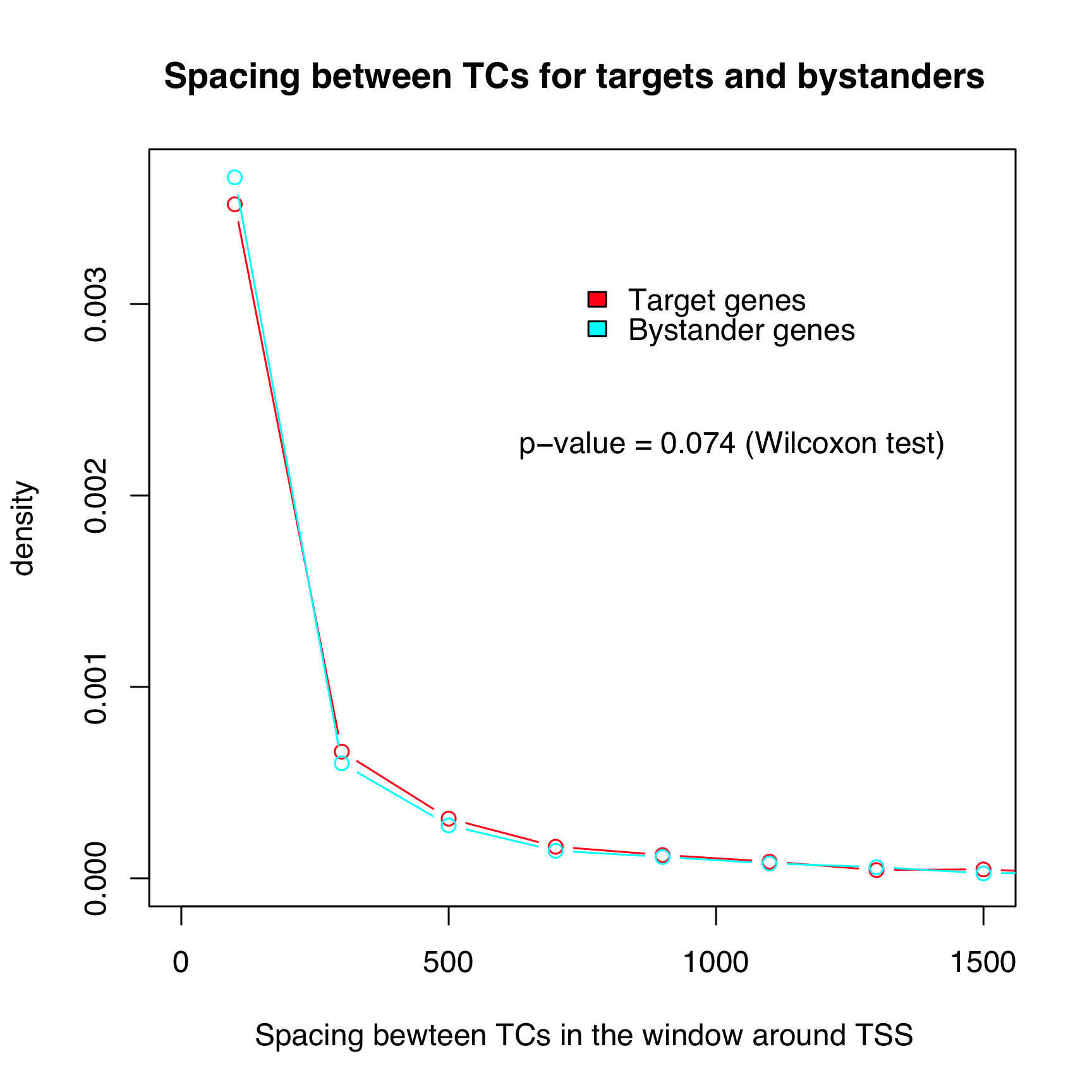


Figure S3. Distance between adjacent TCs for targets and bystanders

The distance between TCs of target genes were not significantly different from the distances of bystander associated TCs.


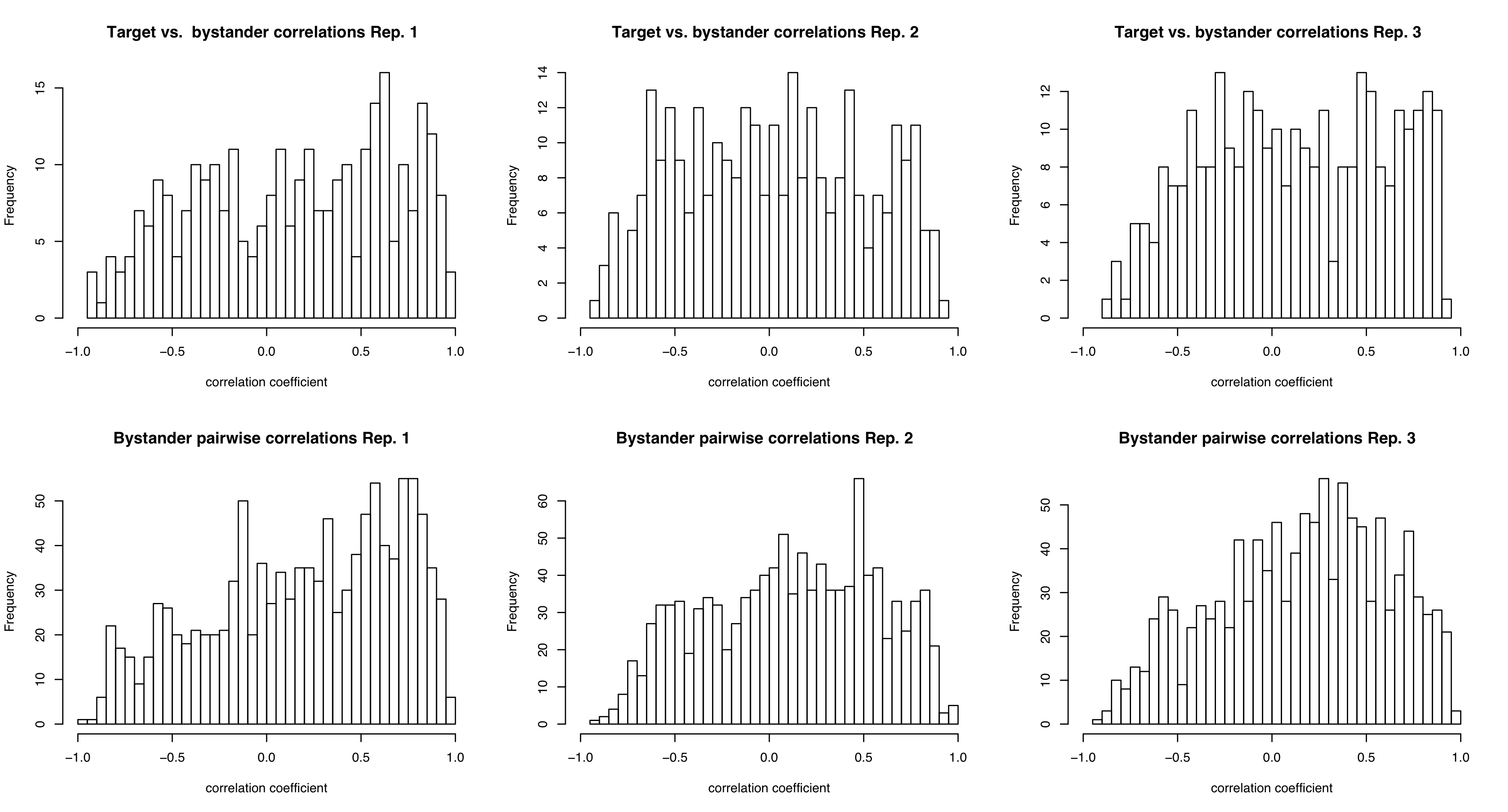


Figure S4. Histograms for Illumina expression correlation coefficients for each replicate experiment

The top row of plots shows the correlation coefficients for expression profiles of all target-bystander gene pairs in all GRBs in the three replicate experiments. The second row shows bystander vs. bystander correlation histograms for each replicate. In this case, every possible bystander-bystander pairing (unless they share bi-directional promoters) in a GRB was considered and correlation coefficients calculated accordingly.


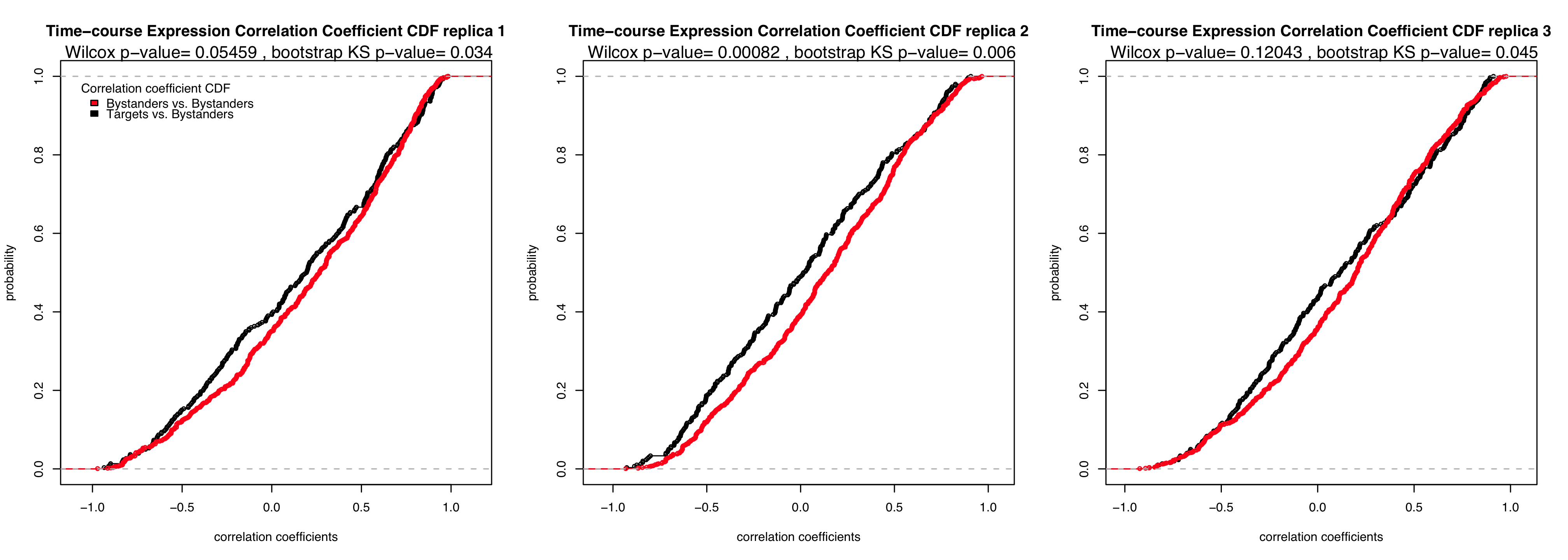


Figure S5. Cumulative distribution plot for Illumina expression correlation coefficients for each replicate experiment

The cumulative distribution of correlation coefficients for expression profiles of bystander pairs in the GRBs (red) and correlation coefficients of expression profiles of target-bystander gene pairs (black) in the GRBs across three replicates.


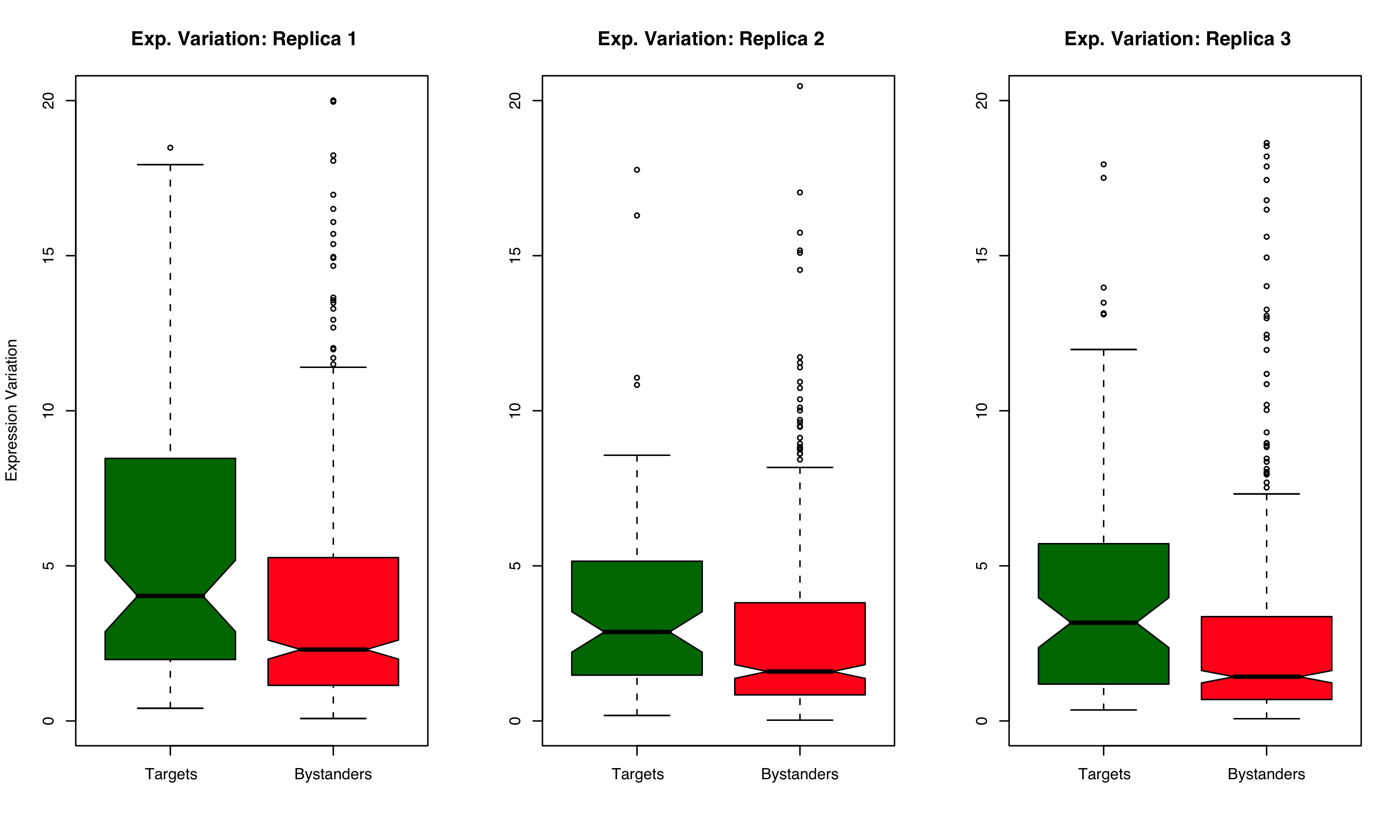
­­­­

Figure S6. Box-plots for expression variation for target and bystander genes

Expression variation for target and bystander genes across three time-course replicates. In all replicates, target genes have higher median variation than bystander genes.

## Supplementary Tables

## Table S1. Over-represented motifs when taking top-level promoters fully covered by CpG islands into account.

|  |  | Background: bystander gene promoters | | Background: Other CpG island promoters | |
| --- | --- | --- | --- | --- | --- |
| Family | Name | Hit  *p*-value | Sequence occurrence *p*-value | Hit  *p*-value | Sequence occurrence *p*-value |
| FORKHEAD | Foxd3 | 0.0001 | 0.0001 | 0.0003 | 0.0001 |
| FORKHEAD | FOXI1 | 0.0001 | 0.0001 | 0.0059 | 0.0001 |
| FORKHEAD | FOXL1 | 0.0067 | 0.0001 | 0.0001 | 0.0001 |
| FORKHEAD | Foxq1 | 0.0001 | 0.0001 | 0.0025 | 0.0001 |
| HOMEO | LHX3 | 0.0001 | 0.0001 | 0.0001 | 0.0001 |
| HOMEO | Lhx3 | 0.0006 | 0.0006 | 0.0016 | 0.0001 |
| MADS | MEF2A | 0.0007 | 0.0005 | 0.0366 | 0.0001 |
| HOMEO | Nkx2-5 | 0.0001 | 0.0001 | 0.0001 | 0.0001 |
| HOMEO | Nobox | 0.0001 | 0.0001 | 0.0001 | 0.0001 |
| HOMEO | Prrx2 | 0.0035 | 0.0001 | 0.0039 | 0.0002 |
| HMG | Sox5 | 0.0001 | 0.0001 | 0.0001 | 0.0001 |
| HMG | SRY | 0.0001 | 0.0001 | 0.0005 | 0.0001 |

The TFBS content of target gene promoters was compared to two different background sets. The promoters used in this analysis were fully covered by CpG islands. The TFBS over-represented in both comparisons are shown. Two distinct *p*-values represent the significance of the number of profile hits in the target set (hit *p*-value) and the significance of the number of sequences having at least one hit in the target set (sequence occurrence *p*-value).

Table S2. Over-represented motifs according to Clover software

| Name | *p*-value (bystanders) | *p*-value (other CpG genes) |
| --- | --- | --- |
| GATA3 | 0 | 0.002 |
| LHX3 | 0 | 0.004 |
| NFYA | 0 | 0 |
| Nkx2-5 | 0 | 0 |
| Pax4 | 0 | 0 |
| SRY | 0.001 | 0 |
| FOXL1 | 0.002 | 0 |
| Gfi | 0.002 | 0 |
| Pdx1 | 0.002 | 0.008 |
| Prrx2 | 0.002 | 0.002 |
| IRF1 | 0.004 | 0.008 |
| Nobox | 0.006 | 0.018 |
| NKX3-1 | 0.007 | 0.048 |
| IRF2 | 0.013 | 0.014 |
| STAT1 | 0.021 | 0.046 |
| Roaz | 0.025 | 0.004 |
| En1 | 0.026 | 0.03 |
| RUSH1-alfa | 0.026 | 0.046 |
| RORA_2 | 0.034 | 0.007 |
| Foxq1 | 0.044 | 0 |

The TFBS content of target gene promoters was compared to two different background sets using Clover. The TFBS over-represented with a *p-*value <= 0.05 in both comparisons are shown.

Table S3. Over-represented motifs according to phylogenetic footprinting analysis

|  | Background: Bystander gene promoters | | Background: Other CpG island gene promoters | |
| --- | --- | --- | --- | --- |
| Motif | Hit *p*-value | Sequence  *p*-value | Hit *p*-value | Sequence *p*-value |
| Arnt | 1.00E-04 | 1.00E-04 | 1.00E-04 | 1.00E-04 |
| Arnt-Ahr | 1.00E-04 | 1.00E-04 | 1.00E-04 | 1.00E-04 |
| Bapx1 | 1.00E-04 | 1.00E-04 | 1.00E-04 | 1.00E-04 |
| ELF5 | 1.00E-04 | 1.00E-04 | 1.00E-04 | 1.00E-04 |
| ELK1 | 1.00E-04 | 1.00E-04 | 1.00E-04 | 1.00E-04 |
| En1 | 1.00E-04 | 1.00E-04 | 1.00E-04 | 1.00E-04 |
| ETS1 | 1.00E-04 | 1.00E-04 | 1.00E-04 | 1.00E-04 |
| FOXC1 | 1.00E-04 | 1.00E-04 | 1.00E-04 | 1.00E-04 |
| FOXL1 | 1.00E-04 | 1.00E-04 | 1.00E-04 | 1.00E-04 |
| Gata1 | 1.00E-04 | 1.00E-04 | 1.00E-04 | 1.00E-04 |
| GATA2 | 1.00E-04 | 1.00E-04 | 1.00E-04 | 1.00E-04 |
| GATA3 | 1.00E-04 | 1.00E-04 | 1.00E-04 | 1.00E-04 |
| Klf4 | 1.00E-04 | 1.00E-04 | 1.00E-04 | 1.00E-04 |
| Mafb | 1.00E-04 | 1.00E-04 | 1.00E-04 | 1.00E-04 |
| Myb | 1.00E-04 | 1.00E-04 | 1.00E-04 | 1.00E-04 |
| MZF1_1-4 | 1.00E-04 | 1.00E-04 | 1.00E-04 | 1.00E-04 |
| MZF1_5-13 | 1.00E-04 | 1.00E-04 | 1.00E-04 | 1.00E-04 |
| Nkx2-5 | 1.00E-04 | 1.00E-04 | 1.00E-04 | 1.00E-04 |
| Pax2 | 1.00E-04 | 1.00E-04 | 1.00E-04 | 1.00E-04 |
| Pdx1 | 1.00E-04 | 1.00E-04 | 1.00E-04 | 1.00E-04 |
| RUSH1-alfa | 1.00E-04 | 1.00E-04 | 1.00E-04 | 1.00E-04 |
| Sox17 | 1.00E-04 | 1.00E-04 | 1.00E-04 | 1.00E-04 |
| Sox5 | 1.00E-04 | 1.00E-04 | 1.00E-04 | 1.00E-04 |
| SP1 | 1.00E-04 | 1.00E-04 | 1.00E-04 | 1.00E-04 |
| SPI1 | 1.00E-04 | 1.00E-04 | 1.00E-04 | 1.00E-04 |
| SPIB | 1.00E-04 | 1.00E-04 | 1.00E-04 | 1.00E-04 |
| TCF11-MafG | 1.00E-04 | 1.00E-04 | 1.00E-04 | 1.00E-04 |
| TFAP2A | 1.00E-04 | 1.00E-04 | 1.00E-04 | 1.00E-04 |
| YY1 | 1.00E-04 | 1.00E-04 | 1.00E-04 | 1.00E-04 |
| ZEB1 | 1.00E-04 | 1.00E-04 | 1.00E-04 | 1.00E-04 |
| ZNF354 | 1.00E-04 | 1.00E-04 | 1.00E-04 | 1.00E-04 |

The TFBS content of target gene promoters was compared to two different background sets using phylogenetic footprinting over-representation analysis. The motifs over-represented in both comparisons, and occurring on at least 50% of the promoter alignments are shown. Two distinct *p*-values represent the significance of the number of profile hits in the target set (hit *p*-value) and the significance of the number of sequences having at least one hit in the target set (sequence occurrence *p*-value).

Table S4. *P*-values for Wilcoxon tests for CpG island characteristics of target genes, bystander genes, other Transcription factors and other CpG island genes

|  | Bystander genes | Other TF genes | Other CpG island genes |
| --- | --- | --- | --- |
| Total CpG island length | *p*-value < 2.2e-16 | *p*-value < 2.2e-16 | *p*-value < 2.2e-16 |
| Number of CpG islands | *p*-value < 2.2e-16 | *p*-value=1.981e-13 | *p*-value=2.220e-16 |
| CpG island length to Gene length ratio | *p*-value < 2.2e-16 | *p*-value < 2.2e-16 | *p*-value < 2.2e-16 |

Results of two-sided Wilcoxon tests between CpG island distributions of the target gene set and 3 different background sets (bystanders, other TFs and other CpG genes) are shown. The cumulative distributions for the compared sets are shown in Figure 6.

## Table S5. Count of expressed and unexpressed bystander genes, conditioned on target gene expression.

|  | # of expressed bystander genes | # of unexpressed bystander genes |
| --- | --- | --- |
| Target gene expressed | 290 | 126 |
| Target gene unexpressed | 237 | 126 |

The number of expressed bystander genes, grouped by the expression status of the corresponding target gene in the GRB. Each counted gene was expressed in at least one time point. The number of expressed and non-expressed bystanders was not significantly associated with expression of targets (*p*-value=0.1928, two-sided Fisher test).

## Table S6. Association between promoter acetylation and target gene expression.

|  | # with promoter ACi | # without promoter ACi |
| --- | --- | --- |
| Expressed target genes | 40 | 44 |
| Unexpressed target genes | 7 | 113 |

Count of the number of expressed and unexpressed target genes at 0h and 96h in the time-course, grouped by promoter acetylation status. Promoter acetylation was significantly associated with target gene expression (*p*-value= 2.357e-12, two-sided Fisher test)

## Table S7. Association between promoter acetylation and bystander gene expression.

|  | # with promoter ACi | # without promoter ACi |
| --- | --- | --- |
| Expressed bystander genes | 319 | 131 |
| Unexpressed bystander genes | 38 | 183 |

The number of expressed bystander genes at 0h and 96h in the time-course experiment, grouped by promoter acetylation status. Promoter acetylation was significantly associated with bystander gene expression (*p*-value < 2.2e-16, two-sided Fisher test)

## Table S8. Association between human:zebrafish HCNE acetylation in a GRB and expression of the corresponding target gene

|  | # with HCNE ACi in the GRB | # without HCNE ACi in the GRB |
| --- | --- | --- |
| # GRBs with an expressed target gene | 12 | 35 |
| # GRBs with no expressed target gene | 3 | 67 |

The number of GRBs of expressed target genes, grouped by the presence of at least one acetylated human:zebrafish HCNE in the GRB. Target gene expression is significantly associated with the presence of an acetylated HCNE (*p*-value = 0.001250, two-sided Fisher test).

## Table S9. Association between human:chicken HCNE acetylation in a GRB and expression of the corresponding target gene

|  | # with HCNE ACi in the GRB | # without HCNE ACi in the GRB |
| --- | --- | --- |
| # GRBs with an expressed target gene | 14 | 33 |
| # GRBs with no expressed target gene | 4 | 66 |

Similar to Supplementary table 8, but using human:chicken HCNEs. Target gene expression is significantly associated with the presence of an acetylated HCNE (*p*-value= 0.0005741, two-sided Fisher test).

Table S10. Association between human:zebrafish HCNE acetylation in a GRB and expression of the bystander genes

|  | # with HCNE ACi in the GRB | # without HCNE ACi in the GRB |
| --- | --- | --- |
| # Expressed bystander genes | 51 | 252 |
| # Unexpressed bystander genes | 15 | 133 |

The number of expressed bystander genes, grouped by the presence of at least one acetylated human:zebrafish HCNE in the GRB. Bystander gene expression is not significantly associated with the presence of an acetylated HCNE (*p*-value = 0.06547, two-sided Fisher test).

Table S11. Association between human:chicken HCNE acetylation in a GRB and expression of the bystander genes

|  | # with HCNE ACi in the GRB | # without HCNE ACi in the GRB |
| --- | --- | --- |
| # Expressed bystander genes | 40 | 263 |
| # Unexpressed bystander genes | 12 | 136 |

Similar to Supplementary table 10, but using human:chicken HCNEs. Bystander gene expression is not significantly associated with the presence of an acetylated HCNE (*p*-value= 0.119, two-sided Fisher test).
